# Supplementary material for: Safety of Four COVID-19 Vaccines across Primary Doses 1, 2, 3 and Booster: A Prospective Cohort Study of Australian Community Pharmacy Vaccinations
Source: Vaccines (Basel). 2022 Nov 25;10(12):2017. doi: 10.3390/vaccines10122017 (PMC9786585; doi:10.3390/vaccines10122017)
Supplement: Supplementary file 1 [file vaccines-10-02017-s001.zip › Table S1 - Survey.docx]

# Questions in day 3 survey

| **Survey section** | **Question** | **Response options** |
| --- | --- | --- |
| Any adverse event | Did you have any reactions following your most recent COVID- 19 vaccination? | 1. Yes  0. No |
| Medical advice/care sought | Did any of the symptoms cause you to seek advice/care from a  doctor/healthcare professional? | 1. Yes  0. No |
|  | If yes, please select the type of advice/care that you sought | 1, Phone advice (e.g. HealthDirect) 2, Care from a GP or Aboriginal  Healthcare Worker (in person, telehealth, email, urgent care clinic, home visit)  3, Visit to a hospital emergency department |
| Solicited reactions | Please select all the reactions that you experienced: | |
|  | Local reaction (pain, redness, swelling, itching at or near the injection site) | 1. Yes  0. No |
|  | Please select all that apply | 1, Pain  2, Redness  3, Swelling  4, Itching |
|  | Please indicate location of rash (select all that apply) | 1, Face  2, Body  3, Arms  4, Legs |
|  | Fever | 1. Yes  0. No |
|  | Please indicate location of rash (select all that apply) | 1, Face  2, Body  3, Arms  4, Legs |
|  | Rash (not at injection site) | 1. Yes  0. No |
|  | Please indicate location of rash (select all that apply) | 1, Face  2, Body  3, Arms  4, Legs |
|  | Did you experience any of the following symptoms at the same time as the rash? | 1, Cough  2, Shortness of breath  3, Noisy or difficult breathing 4, Lip or tongue swelling  0, None of these |
|  | When did the rash after vaccination start? | 1, Within 1 hour after vaccination  2, 1 to 6 hours  3, 7 to 72 hours  4, More than 72 hours (3 days) |
|  | How long did the rash last? | 1, Less than 30 minutes  2, 30 minutes to 24 hours  3, More than 24 hours |
|  | Chills (shivering and feeling cold) | 1. Yes  0. No |
|  | Headache, muscle/body aches, or joint aches/pain | 1. Yes  0. No |
|  | Please select all that apply | 1, Headache  2, Muscle/body aches  3, Joint aches/pain |

| **Survey section** | **Question** | **Response options** |
| --- | --- | --- |
|  | Gastrointestinal symptoms | 1. Yes  0. No |
|  | Please select all that apply | 1, Nausea  2, Vomiting  3, Diarrhoea  4, Abdominal pain |
|  | Fatigue or tiredness | 1. Yes  0. No |
|  | Fainting/loss of consciousness | 1. Yes  0. No |
|  | Did you experience any of the following symptoms around the same time as the faint? (select all that apply) | 1, Cough  2, Shortness of breath  3, Noisy or difficult breathing 4, Lip or tongue swelling  0, None of these |
|  | When did the fainting/loss of  consciousness after vaccination start? | 1, Less than 5 minutes after vaccination 2, 5 to 30 minutes  3, More than 30 minutes |
|  | How long did the fainting/loss of consciousness last? | 1, Less than 1 minute  2, 1 to 5 minutes  3, More than 5 minutes |
|  | Seizure | 1. Yes  0. No |
|  | When did the seizure after vaccination start? | 1, Less than 30 minutes after vaccination 2, Same day  3, Next day  4, 2 or more days after vaccination |
|  | How long did the seizure last? | 1, Less than 1 minute  2, 1 to 5 minutes  3, 6 to 15 minutes  4, 16 to 30 minutes  5, More than 30 minutes |
|  | Did you experience any other symptoms not listed above? | 1. Yes  0. No |
|  | Please specify | Free text |
| Symptom management | Did you take pain or fever medicine (e.g. paracetamol or ibuprofen) *at the time of* vaccination? | 1. Yes  0. No |
|  | Did you use something *after*  vaccination to help your symptoms? | 1. Yes  0. No |
|  | Please check all that apply | 1, Pain/fever relief (e.g. paracetamol or ibuprofen)  2, On skin (e.g. cream, icepack) 3, Anti-allergy medicine (e.g. antihistamine)  4, Other |
| Symptom resolution | Are you still experiencing any of the symptoms you reported? | 0, No, all of my symptoms have gone 1, Yes, I am still experiencing one or more symptoms |
| Health impact | Did any of the symptoms you reported cause you to miss work,  study or normal daily activities? | 1. Yes  0. No |
|  | How many days did you miss? | 1, Less than 1 day  2, 1 day  3, 2 days  4, 3 or more days |

| **Survey section** | **Question** | **Response options** |
| --- | --- | --- |
| Anaphylaxis history | Do you have a history of anaphylaxis or carry an EpiPen? | 1. Yes  0. No |
|  | What was the trigger? | 1, Food  2, Medicine  3, Vaccine  4, Insect venom  5, Other |
| Underlying medical conditions | Do you have any chronic medical conditions? | 1. Yes  0. No |
|  | Please select all that apply | A, Heart disease (coronary heart disease or failure)  B, Poorly controlled blood pressure C, Diabetes/Sugar  D, Chronic lung disease (not including mild/moderate asthma)  E, Obesity with BMI ≥40 kg/m2 F, Chronic kidney failure  G, Chronic liver disease  H, Cancer (not including blood or bone marrow cancer) diagnosed in the last 12 months  I, Blood cancer (e.g. leukaemia, lymphoma or myelodysplastic syndrome) diagnosed within the last 5 years  J, Currently receiving chemotherapy or radiotherapy  K, Organ transplant recipient on immune suppressive therapy  L, Bone marrow transplant recipient in the last 2 years  M, Neurological condition (e.g. stroke, dementia)  N, Chronic inflammatory conditions (e.g. rheumatoid arthritis, lupus)  O, Primary or acquired immunodeficiency (including HIV)  P, Other |
| **End of survey message:**  Thanks for completing the vaccine safety survey.  If you have any concerns about your/your child’s vaccination or any associated reactions, you should speak to your doctor.  If you reported a reaction and needed care/advice from a GP, Aboriginal Healthcare Worker or hospital, you may be contacted by your doctor or state/territory health department for more details.  Visit the [**AusVaxSafety website**](https://ausvaxsafety.org.au/) for vaccine safety data and information on potential vaccine reactions. | | |

# Questions in day 42 survey

| **Survey section** | **Question** | **Response options** |
| --- | --- | --- |
| Any illness/care sought | Did you have any illness that needed medical attention? | 1. Yes  0. No |
|  | If yes  Please select the type of care that you sought for your illness  (select all that apply) | 1. Care from a GP or specialist  2. Visit to a hospital emergency department  3. Admitted to hospital |
| Diagnosis | What was the diagnosis/es? | Free text |
| COVID-19 infection | Have you tested **positive** for COVID-19 since you received your COVID-19 vaccine? | 1. Yes  0. No |
|  | If yes  when was the positive test collected? | Date |
