# Supplementary material for: Safety of Four COVID-19 Vaccines across Primary Doses 1, 2, 3 and Booster: A Prospective Cohort Study of Australian Community Pharmacy Vaccinations
Source: Vaccines (Basel). 2022 Nov 25;10(12):2017. doi: 10.3390/vaccines10122017 (PMC9786585; doi:10.3390/vaccines10122017)
Supplement: Supplementary file 1 [file vaccines-10-02017-s001.zip › Table S2 - Vaccination characteristics extended.docx]

**Table S2: Extended characteristics of COVID-19 vaccination encounters by vaccine brand and dose number.**

|  | **AstraZeneca** | | | | **Moderna** | | | | **Novavax** | | | | **Pfizer** | | | |
| --- | --- | --- | --- | --- | --- | --- | --- | --- | --- | --- | --- | --- | --- | --- | --- | --- |
|  | **Dose 1** | **Dose 2** | **Dose 3** | **Booster** | **Dose 1** | **Dose 2** | **Dose 3** | **Booster** | **Dose 1** | **Dose 2** | **Dose 3** | **Booster** | **Dose 1** | **Dose 2** | **Dose 3** | **Booster** |
| n | 4076 | 25587 | 156 | 661 | 29870 | 37508 | 2218 | 67637 | 1386 | 1248 | 14 | 681 | 12538 | 11571 | 2889 | 58693 |
| Age group, years |  |  |  |  |  |  |  |  |  |  |  |  |  |  |  |  |
| <12 | 4  (0.1) | 23  (0.1) | 0  (0.0) | 0  (0.0) | 85  (0.3) | 47  (0.1) | 0  (0.0) | 11  (0.0) | 0  (0.0) | 0  (0.0) | 0  (0.0) | 0  (0.0) | 7347  (58.6) | 4927  (42.6) | 10  (0.3) | 159  (0.3) |
| 12-15 | 33  (0.8) | 19  (0.1) | 0  (0.0) | 0  (0.0) | 4581  (15.3) | 6032  (16.1) | 8  (0.4) | 92  (0.1) | 0  (0.0) | 0  (0.0) | 0  (0.0) | 0  (0.0) | 1138  (9.1) | 1339  (11.6) | 14  (0.5) | 161  (0.3) |
| 16-19 | 124  (3.0) | 769  (3.0) | 0  (0.0) | 4  (0.6) | 2651  (8.9) | 3340  (8.9) | 40  (1.8) | 2321  (3.4) | 50  (3.6) | 33  (2.6) | 0  (0.0) | 8  (1.2) | 467  (3.7) | 573  (5.0) | 99  (3.4) | 4495  (7.7) |
| 20-29 | 676  (16.6) | 5826  (22.8) | 11  (7.1) | 59  (8.9) | 6086  (20.4) | 7653  (20.4) | 268  (12.1) | 11900  (17.6) | 249  (18.0) | 196  (15.7) | 1  (7.1) | 108  (15.9) | 1228  (9.8) | 1637  (14.1) | 560  (19.4) | 13073  (22.3) |
| 30-39 | 716  (17.6) | 4588  (17.9) | 26  (16.7) | 82  (12.4) | 5497  (18.4) | 6989  (18.6) | 344  (15.5) | 12689  (18.8) | 325  (23.4) | 274  (22.0) | 0  (0.0) | 120  (17.6) | 945  (7.5) | 1331  (11.5) | 595  (20.6) | 11882  (20.2) |
| 40-49 | 623  (15.3) | 2826  (11.0) | 11  (7.1) | 95  (14.4) | 4284  (14.3) | 5160  (13.8) | 315  (14.2) | 9014  (13.3) | 268  (19.3) | 259  (20.8) | 2  (14.3) | 124  (18.2) | 573  (4.6) | 740  (6.4) | 515  (17.8) | 8186  (13.9) |
| 50-59 | 543  (13.3) | 2990  (11.7) | 31  (19.9) | 107  (16.2) | 3275  (11.0) | 4072  (10.9) | 414  (18.7) | 10932  (16.2) | 238  (17.2) | 208  (16.7) | 3  (21.4) | 128  (18.8) | 381  (3.0) | 499  (4.3) | 433  (15.0) | 8032  (13.7) |
| 60-69 | 832  (20.4) | 5645  (22.1) | 44  (28.2) | 186  (28.1) | 2106  (7.1) | 2637  (7.0) | 458  (20.6) | 12107  (17.9) | 158  (11.4) | 166  (13.3) | 4  (28.6) | 114  (16.7) | 263  (2.1) | 295  (2.5) | 389  (13.5) | 7154  (12.2) |
| 70-79 | 341  (8.4) | 2054  (8.0) | 23  (14.7) | 95  (14.4) | 936  (3.1) | 1139  (3.0) | 276  (12.4) | 6207  (9.2) | 75  (5.4) | 83  (6.7) | 2  (14.3) | 61  (9.0) | 128  (1.0) | 146  (1.3) | 219  (7.6) | 3951  (6.7) |
| 80+ | 184  (4.5) | 847  (3.3) | 10  (6.4) | 33  (5.0) | 369  (1.2) | 439  (1.2) | 95  (4.3) | 2364  (3.5) | 23  (1.7) | 29  (2.3) | 2  (14.3) | 18  (2.6) | 68  (0.5) | 84  (0.7) | 55  (1.9) | 1600  (2.7) |
| Sex |  |  |  |  |  |  |  |  |  |  |  |  |  |  |  |  |
| Female | 1293  (31.7) | 7725  (30.2) | 58  (37.2) | 218  (33.0) | 10850  (36.3) | 12939  (34.5) | 812  (36.6) | 24328  (36.0) | 439  (31.7) | 430  (34.5) | 4  (28.6) | 265  (38.9) | 3745  (29.9) | 3609  (31.2) | 940  (32.5) | 20323  (34.6) |
| Male | 1661  (40.8) | 9628  (37.6) | 49  (31.4) | 205  (31.0) | 11609  (38.9) | 13822  (36.9) | 659  (29.7) | 19635  (29.0) | 353  (25.5) | 327  (26.2) | 3  (21.4) | 211  (31.0) | 3643  (29.1) | 3582  (31.0) | 783  (27.1) | 16014  (27.3) |
| Not recorded | 1122  (27.5) | 8234  (32.2) | 49  (31.4) | 238  (36.0) | 7411  (24.8) | 10747  (28.7) | 747  (33.7) | 23674  (35.0) | 594  (42.9) | 491  (39.3) | 7  (50.0) | 205  (30.1) | 5150  (41.1) | 4380  (37.9) | 1166  (40.4) | 22356  (38.1) |
| State |  |  |  |  |  |  |  |  |  |  |  |  |  |  |  |  |
| ACT | 66  (1.6) | 1120  (4.4) | 0  (0.0) | 4  (0.6) | 391  (1.3) | 390  (1.0) | 6  (0.3) | 769  (1.1) | 0  (0.0) | 0  (0.0) | 0  (0.0) | 4  (0.6) | 297  (2.4) | 211  (1.8) | 9  (0.3) | 775  (1.3) |
| NSW | 735  (18.0) | 9533  (37.3) | 108  (69.2) | 187  (28.3) | 3735  (12.5) | 5185  (13.8) | 1283  (57.8) | 16662  (24.6) | 159  (11.5) | 130  (10.4) | 1  (7.1) | 58  (8.5) | 2211  (17.6) | 1664  (14.4) | 1947  (67.4) | 15439  (26.3) |
| NT | 0  (0.0) | 29  (0.1) | 0  (0.0) | 0  (0.0) | 0  (0.0) | 0  (0.0) | 0  (0.0) | 0  (0.0) | 0  (0.0) | 0  (0.0) | 0  (0.0) | 0  (0.0) | 0  (0.0) | 0  (0.0) | 0  (0.0) | 0  (0.0) |
| QLD | 350  (8.6) | 2231  (8.7) | 10  (6.4) | 74  (11.2) | 5444  (18.2) | 5546  (14.8) | 156  (7.0) | 5894  (8.7) | 233  (16.8) | 165  (13.2) | 1  (7.1) | 36  (5.3) | 951  (7.6) | 944  (8.2) | 104  (3.6) | 3337  (5.7) |
| SA | 228  (5.6) | 878  (3.4) | 7  (4.5) | 18  (2.7) | 2310  (7.7) | 2232  (6.0) | 109  (4.9) | 2449  (3.6) | 123  (8.9) | 109  (8.7) | 1  (7.1) | 55  (8.1) | 707  (5.6) | 604  (5.2) | 56  (1.9) | 1986  (3.4) |
| TAS | 9  (0.2) | 180  (0.7) | 2  (1.3) | 0  (0.0) | 777  (2.6) | 712  (1.9) | 11  (0.5) | 537  (0.8) | 31  (2.2) | 23  (1.8) | 0  (0.0) | 2  (0.3) | 25  (0.2) | 37  (0.3) | 26  (0.9) | 759  (1.3) |
| VIC | 1580  (38.8) | 9127  (35.7) | 23  (14.7) | 261  (39.5) | 8332  (27.9) | 14944  (39.8) | 467  (21.1) | 28523  (42.2) | 481  (34.7) | 425  (34.1) | 9  (64.3) | 313  (46.0) | 4699  (37.5) | 4174  (36.1) | 590  (20.4) | 26404  (45.0) |
| WA | 1108  (27.2) | 2489  (9.7) | 6  (3.8) | 117  (17.7) | 8881  (29.7) | 8499  (22.7) | 186  (8.4) | 12803  (18.9) | 359  (25.9) | 396  (31.7) | 2  (14.3) | 213  (31.3) | 3648  (29.1) | 3937  (34.0) | 157  (5.4) | 9993  (17.0) |
| Chronic medical condition/s  ^a^ | 271  (15.6) | 1163  (12.4) | 19  (22.6) | 62  (20.4) | 1328  (9.7) | 1243  (8.9) | 357  (31.1) | 4051  (12.8) | 100  (15.6) | 84  (15.7) | 3  (50.0) | 63  (19.4) | 260  (4.5) | 221  (5.0) | 300  (21.4) | 2716  (10.9) |
| Blood cancer within 5 years | 3  (1.1) | 31  (2.7) | 0  (0.0) | 2  (3.3) | 29  (2.2) | 25  (2.0) | 27  (7.6) | 90  (2.3) | 1  (1.0) | 1  (1.2) | 0  (0.0) | 4  (6.3) | 6  (2.4) | 8  (3.6) | 20  (6.7) | 50  (1.9) |
| Bone marrow transplant within 2 years | 0  (0.0) | 1  (0.1) | 0  (0.0) | 1  (1.6) | 2  (0.2) | 3  (0.2) | 1  (0.3) | 1  (0.0) | 0  (0.0) | 0  (0.0) | 0  (0.0) | 0  (0.0) | 1  (0.4) | 3  (1.4) | 1  (0.3) | 2  (0.1) |
| Cancer (excl. blood or bone marrow) within 12 months | 11  (4.1) | 54  (4.7) | 2  (10.5) | 2  (3.3) | 47  (3.6) | 36  (2.9) | 45  (12.7) | 165  (4.1) | 1  (1.0) | 3  (3.6) | 0  (0.0) | 2  (3.2) | 11  (4.3) | 8  (3.6) | 32  (10.7) | 126  (4.7) |
| Currently receiving chemotherapy or radiotherapy | 6  (2.2) | 23  (2.0) | 1  (5.3) | 2  (3.3) | 21  (1.6) | 18  (1.5) | 37  (10.4) | 58  (1.5) | 1  (1.0) | 2  (2.4) | 0  (0.0) | 1  (1.6) | 8  (3.1) | 7  (3.2) | 28  (9.4) | 33  (1.2) |
| Chronic inflammatory conditions | 51  (19.0) | 196  (17.1) | 7  (36.8) | 10  (16.4) | 242  (18.4) | 211  (17.2) | 92  (25.9) | 644  (16.1) | 18  (18.4) | 22  (26.5) | 0  (0.0) | 14  (22.2) | 33  (12.9) | 33  (14.9) | 86  (28.8) | 429  (16.0) |
| Chronic kidney failure | 4  (1.5) | 25  (2.2) | 3  (15.8) | 2  (3.3) | 16  (1.2) | 13  (1.1) | 17  (4.8) | 94  (2.4) | 0  (0.0) | 0  (0.0) | 0  (0.0) | 1  (1.6) | 7  (2.7) | 6  (2.7) | 8  (2.7) | 67  (2.5) |
| Chronic liver disease | 5  (1.9) | 18  (1.6) | 0  (0.0) | 2  (3.3) | 21  (1.6) | 27  (2.2) | 8  (2.3) | 74  (1.9) | 1  (1.0) | 1  (1.2) | 0  (0.0) | 0  (0.0) | 1  (0.4) | 0  (0.0) | 5  (1.7) | 52  (1.9) |
| Chronic lung disease | 33  (12.3) | 133  (11.6) | 2  (10.5) | 5  (8.2) | 92  (7.0) | 100  (8.1) | 39  (11.0) | 359  (9.0) | 7  (7.1) | 11  (13.3) | 0  (0.0) | 8  (12.7) | 23  (9.0) | 19  (8.6) | 22  (7.4) | 214  (8.0) |
| Diabetes | 59  (21.9) | 329  (28.6) | 5  (26.3) | 17  (27.9) | 293  (22.3) | 294  (23.9) | 48  (13.5) | 1112  (27.8) | 15  (15.3) | 20  (24.1) | 1  (33.3) | 10  (15.9) | 30  (11.8) | 30  (13.6) | 54  (18.1) | 767  (28.6) |
| Heart disease | 38  (14.1) | 205  (17.8) | 4  (21.1) | 14  (23.0) | 143  (10.9) | 130  (10.6) | 44  (12.4) | 554  (13.9) | 9  (9.2) | 11  (13.3) | 1  (33.3) | 12  (19.0) | 13  (5.1) | 23  (10.4) | 17  (5.7) | 394  (14.7) |
| Primary or acquired immunodeficiency | 1  (0.4) | 8  (0.7) | 0  (0.0) | 0  (0.0) | 21  (1.6) | 20  (1.6) | 17  (4.8) | 49  (1.2) | 2  (2.0) | 1  (1.2) | 0  (0.0) | 0  (0.0) | 6  (2.4) | 3  (1.4) | 16  (5.4) | 31  (1.2) |
| Neurological condition | 12  (4.5) | 51  (4.4) | 2  (10.5) | 3  (4.9) | 72  (5.5) | 55  (4.5) | 12  (3.4) | 192  (4.8) | 9  (9.2) | 6  (7.2) | 0  (0.0) | 5  (7.9) | 14  (5.5) | 10  (4.5) | 14  (4.7) | 143  (5.3) |
| Obesity | 25  (9.3) | 108  (9.4) | 1  (5.3) | 4  (6.6) | 141  (10.7) | 113  (9.2) | 24  (6.8) | 419  (10.5) | 8  (8.2) | 11  (13.3) | 0  (0.0) | 7  (11.1) | 23  (9.0) | 16  (7.2) | 24  (8.0) | 279  (10.4) |
| Other | 126  (46.8) | 397  (34.6) | 7  (36.8) | 19  (31.1) | 590  (45.0) | 545  (44.4) | 96  (27.0) | 1452  (36.3) | 54  (55.1) | 34  (41.0) | 1  (33.3) | 24  (38.1) | 133  (52.2) | 102  (46.2) | 79  (26.4) | 929  (34.7) |
| Organ transplant recipient on immune suppressive therapy | 0  (0.0) | 6  (0.5) | 3  (15.8) | 0  (0.0) | 13  (1.0) | 6  (0.5) | 12  (3.4) | 34  (0.9) | 0  (0.0) | 0  (0.0) | 0  (0.0) | 1  (1.6) | 4  (1.6) | 2  (0.9) | 11  (3.7) | 28  (1.0) |
| Poorly controlled blood pressure | 19  (7.1) | 106  (9.2) | 2  (10.5) | 3  (4.9) | 119  (9.1) | 84  (6.8) | 19  (5.4) | 338  (8.5) | 6  (6.1) | 4  (4.8) | 1  (33.3) | 5  (7.9) | 17  (6.7) | 8  (3.6) | 11  (3.7) | 220  (8.2) |
| History of anaphylaxis | 51  (2.9) | 140  (1.5) | 4  (4.8) | 12  (3.9) | 331  (2.4) | 321  (2.3) | 27  (2.3) | 624  (2.0) | 20  (3.1) | 15  (2.8) | 0  (0.0) | 13  (4.0) | 105  (1.8) | 99  (2.2) | 18  (1.3) | 444  (1.8) |
| Pain/fever medicine pre vaccination | 597  (34.5) | 1793  (19.1) | 13  (15.5) | 48  (15.8) | 3082  (22.6) | 5750  (41.2) | 332  (28.9) | 10235  (32.4) | 111  (17.3) | 120  (22.4) | 3  (50.0) | 61  (18.8) | 1322  (22.7) | 1087  (24.4) | 381  (27.2) | 7189  (28.8) |
| Day 3 Survey |  |  |  |  |  |  |  |  |  |  |  |  |  |  |  |  |
| Sent | 3901  (95.7) | 25407  (99.3) | 154  (98.7) | 650  (98.3) | 29489  (98.7) | 37354  (99.6) | 2133  (96.2) | 66326  (98.1) | 1338  (96.5) | 1214  (97.3) | 11  (78.6) | 614  (90.2) | 12398  (98.9) | 11345  (98.0) | 2776  (96.1) | 57362  (97.7) |
| Responded | 1732  (44.4) | 9410  (37.0) | 84  (54.5) | 304  (46.8) | 13647  (46.3) | 13944  (37.3) | 1149  (53.9) | 31592  (47.6) | 643  (48.1) | 536  (44.2) | 6  (54.5) | 324  (52.8) | 5818  (46.9) | 4460  (39.3) | 1399  (50.4) | 24976  (43.5) |
| Reported adverse event | 1010  (58.3) | 2196  (23.3) | 19  (22.6) | 71  (23.4) | 5423  (39.7) | 9120  (65.4) | 704  (61.3) | 17292  (54.7) | 222  (34.5) | 315  (58.8) | 0  (0.0) | 93  (28.7) | 1516  (26.1) | 1408  (31.6) | 587  (42.0) | 10391  (41.6) |
| Medication to relieve symptoms | 634  (63.0) | 1183  (54.2) | 13  (68.4) | 35  (49.3) | 2555  (47.4) | 6089  (67.1) | 446  (63.6) | 10497  (61.0) | 110  (50.0) | 185  (59.3) | 0  (0.0) | 46  (49.5) | 724  (48.0) | 774  (55.3) | 313  (53.5) | 5874  (56.9) |
| Reported medical review or advice ^b^ | 81  (4.7) | 107  (1.1) | 0  (0.0) | 6  (2.0) | 225  (1.6) | 648  (4.6) | 13  (1.1) | 554  (1.8) | 21  (3.3) | 16  (3.0) | 0  (0.0) | 12  (3.7) | 57  (1.0) | 73  (1.6) | 15  (1.1) | 344  (1.4) |
| Care from a GP | 38  (48.7) | 52  (59.8) | 0  (0.0) | 1  (25.0) | 97  (48.0) | 294  (49.9) | 5  (71.4) | 221  (49.7) | 10  (55.6) | 9  (56.2) | 0  (0.0) | 4  (36.4) | 26  (53.1) | 28  (43.1) | 8  (72.7) | 144  (51.4) |
| Emergency department visit | 15  (19.2) | 16  (18.4) | 0  (0.0) | 1  (25.0) | 45  (22.3) | 119  (20.2) | 1  (14.3) | 71  (16.0) | 3  (16.7) | 2  (12.5) | 0  (0.0) | 2  (18.2) | 12  (24.5) | 12  (18.5) | 0  (0.0) | 41  (14.6) |
| Phone advice | 36  (46.2) | 30  (34.5) | 0  (0.0) | 2  (50.0) | 86  (42.6) | 238  (40.4) | 2  (28.6) | 184  (41.3) | 8  (44.4) | 8  (50.0) | 0  (0.0) | 5  (45.5) | 21  (42.9) | 29  (44.6) | 3  (27.3) | 111  (39.6) |
| Day 42 Survey |  |  |  |  |  |  |  |  |  |  |  |  |  |  |  |  |
| Sent | 1731  (42.5) | 9399  (36.7) | 82  (52.6) | 236  (35.7) | 13600  (45.5) | 13875  (37.0) | 1052  (47.4) | 27656  (40.9) | 469  (33.8) | 184  (14.7) | 0  (0.0) | 45  (6.6) | 5654  (45.1) | 2856  (24.7) | 1269  (43.9) | 20652  (35.2) |
| Responded | 999  (57.7) | 5487  (58.4) | 47  (57.3) | 151  (64.0) | 7482  (55.0) | 7574  (54.6) | 687  (65.3) | 15976  (57.8) | 300  (64.0) | 150  (81.5) | 0  (0.0) | 35  (77.8) | 2911  (51.5) | 1616  (56.6) | 779  (61.4) | 11135  (53.9) |
| Reported new illness | 40  (4.0) | 113  (2.1) | 2  (4.3) | 5  (3.3) | 192  (2.6) | 213  (2.8) | 20  (2.9) | 300  (1.9) | 19  (6.3) | 3  (2.0) | 0  (0.0) | 3  (8.6) | 62  (2.1) | 33  (2.0) | 13  (1.7) | 164  (1.5) |
| Tested COVID-19 positive | 9  (0.9) | 36  (0.7) | 3  (6.8) | 3  (4.3) | 24  (0.3) | 72  (1.0) | 27  (4.4) | 273  (2.8) | 0  (0.0) | 0  (0.0) | 0  (0.0) | 0  (0.0) | 199  (7.6) | 37  (5.1) | 29  (4.5) | 219  (3.4) |

All reported as n (%)

Questions on ‘health impact’ (in the day 3 survey) are not included in this analysis

^a^ Respondents could select multiple chronic medical conditions. Proportions add to more than 100%.

^b^ Respondents could select multiple levels of medical review or advice. This shows engagement with the health system, not the highest level of care sought. Proportions add to more than 100%.
