# Supplementary material for: Safety of Four COVID-19 Vaccines across Primary Doses 1, 2, 3 and Booster: A Prospective Cohort Study of Australian Community Pharmacy Vaccinations
Source: Vaccines (Basel). 2022 Nov 25;10(12):2017. doi: 10.3390/vaccines10122017 (PMC9786585; doi:10.3390/vaccines10122017)
Supplement: Supplementary file 1 [file vaccines-10-02017-s001.zip › Table S3 - Extended characteristics Day 3 medical review or advice.docx]

**Table S3: Extended characteristics of COVID-19 vaccination encounters for people reporting medical review or advice at day 3 by vaccine brand and dose number.**

|  | **AstraZeneca** | | | | **Moderna** | | | | **Novavax** | | | | **Pfizer** | | | |
| --- | --- | --- | --- | --- | --- | --- | --- | --- | --- | --- | --- | --- | --- | --- | --- | --- |
|  | **Dose 1** | **Dose 2** | **Dose 3** | **Booster** | **Dose 1** | **Dose 2** | **Dose 3** | **Booster** | **Dose 1** | **Dose 2** | **Dose 3** | **Booster** | **Dose 1** | **Dose 2** | **Dose 3** | **Booster** |
| n | 81 | 107 | 0 | 6 | 225 | 648 | 13 | 554 | 21 | 16 | 0 | 12 | 57 | 73 | 15 | 344 |
| Age group, years |  |  | - |  |  |  |  |  |  |  | - |  |  |  |  |  |
| <12 | 0  (0.0) | 0  (0.0) | - | 0  (0.0) | 1  (0.4) | 0  (0.0) | 0  (0.0) | 0  (0.0) | 0  (0.0) | 0  (0.0) | - | 0  (0.0) | 14  (24.6) | 17  (23.3) | 0  (0.0) | 0  (0.0) |
| 12-15 | 0  (0.0) | 0  (0.0) | - | 0  (0.0) | 24  (10.7) | 84  (13.0) | 0  (0.0) | 1  (0.2) | 0  (0.0) | 0  (0.0) | - | 0  (0.0) | 4  (7.0) | 4  (5.5) | 0  (0.0) | 0  (0.0) |
| 16-19 | 1  (1.2) | 2  (1.9) | - | 0  (0.0) | 17  (7.6) | 38  (5.9) | 1  (7.7) | 9  (1.6) | 1  (4.8) | 0  (0.0) | - | 0  (0.0) | 3  (5.3) | 2  (2.7) | 1  (6.7) | 22  (6.4) |
| 20-29 | 14  (17.3) | 22  (20.6) | - | 0  (0.0) | 50  (22.2) | 121  (18.7) | 1  (7.7) | 98  (17.7) | 4  (19.0) | 2  (12.5) | - | 1  (8.3) | 7  (12.3) | 11  (15.1) | 0  (0.0) | 65  (18.9) |
| 30-39 | 27  (33.3) | 21  (19.6) | - | 0  (0.0) | 62  (27.6) | 162  (25.0) | 1  (7.7) | 121  (21.8) | 9  (42.9) | 8  (50.0) | - | 2  (16.7) | 15  (26.3) | 16  (21.9) | 1  (6.7) | 80  (23.3) |
| 40-49 | 19  (23.5) | 25  (23.4) | - | 4  (66.7) | 30  (13.3) | 111  (17.1) | 3  (23.1) | 102  (18.4) | 1  (4.8) | 1  (6.2) | - | 7  (58.3) | 8  (14.0) | 10  (13.7) | 4  (26.7) | 70  (20.3) |
| 50-59 | 8  (9.9) | 16  (15.0) | - | 1  (16.7) | 29  (12.9) | 80  (12.3) | 3  (23.1) | 113  (20.4) | 6  (28.6) | 5  (31.2) | - | 1  (8.3) | 3  (5.3) | 11  (15.1) | 3  (20.0) | 54  (15.7) |
| 60-69 | 10  (12.3) | 11  (10.3) | - | 1  (16.7) | 10  (4.4) | 40  (6.2) | 3  (23.1) | 76  (13.7) | 0  (0.0) | 0  (0.0) | - | 1  (8.3) | 2  (3.5) | 2  (2.7) | 4  (26.7) | 36  (10.5) |
| 70-79 | 1  (1.2) | 7  (6.5) | - | 0  (0.0) | 1  (0.4) | 10  (1.5) | 1  (7.7) | 28  (5.1) | 0  (0.0) | 0  (0.0) | - | 0  (0.0) | 1  (1.8) | 0  (0.0) | 2  (13.3) | 13  (3.8) |
| 80+ | 1  (1.2) | 3  (2.8) | - | 0  (0.0) | 1  (0.4) | 2  (0.3) | 0  (0.0) | 6  (1.1) | 0  (0.0) | 0  (0.0) | - | 0  (0.0) | 0  (0.0) | 0  (0.0) | 0  (0.0) | 4  (1.2) |
| Sex |  |  | - |  |  |  |  |  |  |  | - |  |  |  |  |  |
| Female | 28  (34.6) | 54  (50.5) | - | 0  (0.0) | 88  (39.1) | 311  (48.0) | 7  (53.8) | 260  (46.9) | 7  (33.3) | 6  (37.5) | - | 5  (41.7) | 16  (28.1) | 24  (32.9) | 8  (53.3) | 175  (50.9) |
| Male | 29  (35.8) | 25  (23.4) | - | 0  (0.0) | 76  (33.8) | 155  (23.9) | 4  (30.8) | 108  (19.5) | 6  (28.6) | 3  (18.8) | - | 4  (33.3) | 20  (35.1) | 20  (27.4) | 3  (20.0) | 52  (15.1) |
| Not recorded | 24  (29.6) | 28  (26.2) | - | 6  (100.0) | 61  (27.1) | 182  (28.1) | 2  (15.4) | 186  (33.6) | 8  (38.1) | 7  (43.8) | - | 3  (25.0) | 21  (36.8) | 29  (39.7) | 4  (26.7) | 117  (34.0) |
| State |  |  | - |  |  |  |  |  |  |  | - |  |  |  |  |  |
| ACT | 0  (0.0) | 2  (1.9) | - | 0  (0.0) | 2  (0.9) | 3  (0.5) | 0  (0.0) | 3  (0.5) | 0  (0.0) | 0  (0.0) | - | 0  (0.0) | 0  (0.0) | 0  (0.0) | 0  (0.0) | 7  (2.0) |
| NSW | 10  (12.3) | 30  (28.0) | - | 1  (16.7) | 19  (8.4) | 75  (11.6) | 6  (46.2) | 113  (20.4) | 5  (23.8) | 1  (6.2) | - | 1  (8.3) | 7  (12.3) | 3  (4.1) | 8  (53.3) | 69  (20.1) |
| QLD | 3  (3.7) | 8  (7.5) | - | 1  (16.7) | 37  (16.4) | 102  (15.7) | 0  (0.0) | 42  (7.6) | 5  (23.8) | 1  (6.2) | - | 1  (8.3) | 5  (8.8) | 9  (12.3) | 1  (6.7) | 16  (4.7) |
| SA | 4  (4.9) | 6  (5.6) | - | 0  (0.0) | 14  (6.2) | 39  (6.0) | 0  (0.0) | 18  (3.2) | 1  (4.8) | 3  (18.8) | - | 0  (0.0) | 7  (12.3) | 7  (9.6) | 0  (0.0) | 14  (4.1) |
| TAS | 0  (0.0) | 0  (0.0) | - | 0  (0.0) | 3  (1.3) | 6  (0.9) | 1  (7.7) | 3  (0.5) | 0  (0.0) | 1  (6.2) | - | 0  (0.0) | 0  (0.0) | 0  (0.0) | 0  (0.0) | 3  (0.9) |
| VIC | 39  (48.1) | 49  (45.8) | - | 4  (66.7) | 90  (40.0) | 295  (45.5) | 5  (38.5) | 269  (48.6) | 4  (19.0) | 4  (25.0) | - | 9  (75.0) | 19  (33.3) | 25  (34.2) | 5  (33.3) | 174  (50.6) |
| WA | 25  (30.9) | 12  (11.2) | - | 0  (0.0) | 60  (26.7) | 128  (19.8) | 1  (7.7) | 106  (19.1) | 6  (28.6) | 6  (37.5) | - | 1  (8.3) | 19  (33.3) | 29  (39.7) | 1  (6.7) | 61  (17.7) |
| Chronic medical condition/s ^a^ | 12  (14.8) | 29  (27.1) | - | 1  (16.7) | 36  (16.0) | 93  (14.4) | 7  (53.8) | 86  (15.5) | 6  (28.6) | 3  (18.8) | - | 1  (8.3) | 7  (12.3) | 12  (16.4) | 4  (26.7) | 64  (18.6) |
| Blood cancer within 5 years | 0  (0.0) | 0  (0.0) | - | 0  (0.0) | 0  (0.0) | 4  (4.3) | 0  (0.0) | 2  (2.4) | 0  (0.0) | 0  (0.0) | - | 0  (0.0) | 0  (0.0) | 0  (0.0) | 0  (0.0) | 1  (1.6) |
| Bone marrow transplant within 2 years | 12  (100.0) | 28  (100.0) | - | 1  (100.0) | 35  (100.0) | 92  (100.0) | 7  (100.0) | 85  (100.0) | 6  (100.0) | 3  (100.0) | - | 1  (100.0) | 7  (100.0) | 12  (100.0) | 4  (100.0) | 63  (100.0) |
| Cancer (excl. blood or bone marrow) within 12 months | 0  (0.0) | 3  (10.7) | - | 0  (0.0) | 1  (2.9) | 2  (2.2) | 0  (0.0) | 4  (4.7) | 0  (0.0) | 0  (0.0) | - | 0  (0.0) | 0  (0.0) | 0  (0.0) | 0  (0.0) | 1  (1.6) |
| Currently receiving chemotherapy or radiotherapy | 0  (0.0) | 0  (0.0) | - | 0  (0.0) | 1  (2.9) | 2  (2.2) | 0  (0.0) | 2  (2.4) | 0  (0.0) | 0  (0.0) | - | 0  (0.0) | 0  (0.0) | 0  (0.0) | 0  (0.0) | 0  (0.0) |
| Chronic inflammatory conditions | 1  (8.3) | 8  (28.6) | - | 0  (0.0) | 8  (22.9) | 14  (15.2) | 1  (14.3) | 24  (28.2) | 0  (0.0) | 0  (0.0) | - | 0  (0.0) | 2  (28.6) | 1  (8.3) | 2  (50.0) | 14  (22.2) |
| Chronic kidney failure | 0  (0.0) | 0  (0.0) | - | 0  (0.0) | 0  (0.0) | 0  (0.0) | 0  (0.0) | 2  (2.4) | 0  (0.0) | 0  (0.0) | - | 0  (0.0) | 0  (0.0) | 0  (0.0) | 1  (25.0) | 0  (0.0) |
| Chronic liver disease | 0  (0.0) | 0  (0.0) | - | 0  (0.0) | 0  (0.0) | 4  (4.3) | 0  (0.0) | 0  (0.0) | 0  (0.0) | 0  (0.0) | - | 0  (0.0) | 0  (0.0) | 0  (0.0) | 0  (0.0) | 2  (3.2) |
| Chronic lung disease | 2  (16.7) | 1  (3.6) | - | 1  (100.0) | 5  (14.3) | 9  (9.8) | 0  (0.0) | 5  (5.9) | 1  (16.7) | 1  (33.3) | - | 1  (100.0) | 0  (0.0) | 1  (8.3) | 0  (0.0) | 5  (7.9) |
| Diabetes | 1  (8.3) | 9  (32.1) | - | 0  (0.0) | 4  (11.4) | 16  (17.4) | 0  (0.0) | 13  (15.3) | 0  (0.0) | 0  (0.0) | - | 0  (0.0) | 1  (14.3) | 2  (16.7) | 1  (25.0) | 19  (30.2) |
| Heart disease | 1  (8.3) | 2  (7.1) | - | 1  (100.0) | 4  (11.4) | 7  (7.6) | 0  (0.0) | 9  (10.6) | 0  (0.0) | 0  (0.0) | - | 0  (0.0) | 0  (0.0) | 1  (8.3) | 0  (0.0) | 8  (12.7) |
| Primary or acquired immunodeficiency | 0  (0.0) | 1  (3.6) | - | 0  (0.0) | 0  (0.0) | 1  (1.1) | 0  (0.0) | 1  (1.2) | 0  (0.0) | 0  (0.0) | - | 0  (0.0) | 0  (0.0) | 0  (0.0) | 0  (0.0) | 1  (1.6) |
| Neurological condition | 1  (8.3) | 0  (0.0) | - | 0  (0.0) | 3  (8.6) | 6  (6.5) | 1  (14.3) | 5  (5.9) | 1  (16.7) | 0  (0.0) | - | 0  (0.0) | 0  (0.0) | 0  (0.0) | 0  (0.0) | 3  (4.8) |
| Obesity | 1  (8.3) | 3  (10.7) | - | 1  (100.0) | 4  (11.4) | 8  (8.7) | 0  (0.0) | 7  (8.2) | 0  (0.0) | 0  (0.0) | - | 1  (100.0) | 1  (14.3) | 0  (0.0) | 0  (0.0) | 7  (11.1) |
| Other | 9  (75.0) | 14  (50.0) | - | 0  (0.0) | 20  (57.1) | 49  (53.3) | 5  (71.4) | 41  (48.2) | 5  (83.3) | 3  (100.0) | - | 0  (0.0) | 5  (71.4) | 7  (58.3) | 1  (25.0) | 24  (38.1) |
| Organ transplant recipient on immune suppressive therapy | 0  (0.0) | 0  (0.0) | - | 0  (0.0) | 0  (0.0) | 0  (0.0) | 0  (0.0) | 1  (1.2) | 0  (0.0) | 0  (0.0) | - | 0  (0.0) | 0  (0.0) | 0  (0.0) | 0  (0.0) | 0  (0.0) |
| Poorly controlled blood pressure | 1  (8.3) | 2  (7.1) | - | 1  (100.0) | 3  (8.6) | 8  (8.7) | 0  (0.0) | 6  (7.1) | 0  (0.0) | 0  (0.0) | - | 0  (0.0) | 0  (0.0) | 1  (8.3) | 0  (0.0) | 6  (9.5) |
| History of anaphylaxis | 2  (2.5) | 7  (6.5) | - | 1  (16.7) | 9  (4.0) | 30  (4.6) | 2  (15.4) | 22  (4.0) | 0  (0.0) | 2  (12.5) | - | 0  (0.0) | 3  (5.3) | 4  (5.5) | 0  (0.0) | 17  (4.9) |
| Day 3 Survey, type of medical review or advice ^b^ |  |  |  |  |  |  |  |  |  |  |  |  |  |  |  |  |
| Care from a GP | 38  (48.7) | 52  (59.8) | - | 1  (25.0) | 97  (48.0) | 294  (49.9) | 5  (71.4) | 221  (49.7) | 10  (55.6) | 9  (56.2) | - | 4  (36.4) | 26  (53.1) | 28  (43.1) | 8  (72.7) | 144  (51.4) |
| Emergency department visit | 15  (19.2) | 16  (18.4) | - | 1  (25.0) | 45  (22.3) | 119  (20.2) | 1  (14.3) | 71  (16.0) | 3  (16.7) | 2  (12.5) | - | 2  (18.2) | 12  (24.5) | 12  (18.5) | 0  (0.0) | 41  (14.6) |
| Phone advice | 36  (46.2) | 30  (34.5) | - | 2  (50.0) | 86  (42.6) | 238  (40.4) | 2  (28.6) | 184  (41.3) | 8  (44.4) | 8  (50.0) | - | 5  (45.5) | 21  (42.9) | 29  (44.6) | 3  (27.3) | 111  (39.6) |

All reported as n (%)

Questions on ‘health impact’ (in the day 3 survey) are not included in this analysis

^a^ Respondents could select multiple chronic medical conditions. Proportions add to more than 100%.

^b^ Respondents could select multiple levels of medical review or advice. This shows engagement with the health system, not the highest level of care sought. Proportions add to more than 100%.
