# Supplementary material for: Safety of Four COVID-19 Vaccines across Primary Doses 1, 2, 3 and Booster: A Prospective Cohort Study of Australian Community Pharmacy Vaccinations
Source: Vaccines (Basel). 2022 Nov 25;10(12):2017. doi: 10.3390/vaccines10122017 (PMC9786585; doi:10.3390/vaccines10122017)
Supplement: Supplementary file 1 [file vaccines-10-02017-s001.zip › Table S4 - Day 42 self reported diagnoses following vaccination.docx]

**Table S4: Self-reported diagnoses on day 42 following a new serious illness requiring medical attendance after COVID-19 vaccination. Diagnoses were coded in accordance with MedDRA®, the Medical Dictionary for Regulatory Activities terminology.**

| **Self-reported diagnosis** | **Count** |
| --- | --- |
| COVID-19 | 66 |
| Other* | 54 |
| Musculoskeletal pain | 36 |
| Awaiting diagnosis* | 36 |
| Chest pain | 36 |
| Vaccine reaction self declared* | 32 |
| Lower respiratory tract infection | 26 |
| Viral infection | 23 |
| Headache | 21 |
| Urinary tract infection | 21 |
| Palpitations | 21 |
| Herpes zoster | 21 |
| Pneumonia | 19 |
| Fatigue | 18 |
| Cardiac disorder | 17 |
| Vertigo | 17 |
| Lymphadenopathy | 17 |
| Pericarditis | 16 |
| Asthma | 16 |
| Tonsillitis | 15 |
| Hypertension | 14 |
| Urticaria | 14 |
| Infection | 14 |
| Rash | 13 |
| Sinusitis | 13 |
| Myocarditis | 13 |
| Influenza | 13 |
| Cellulitis | 11 |
| Migraine | 11 |
| Gastroenteritis | 10 |
| Hypersensitivity | 9 |
| Anxiety | 9 |
| Dyspnoea | 8 |
| Bronchitis | 7 |
| Influenza like illness | 7 |
| Oropharyngeal pain | 7 |
| Inflammation | 7 |
| Atrial fibrillation | 7 |
| Nephrolithiasis | 6 |
| Cholecystitis | 6 |
| Pulmonary embolism | 6 |
| Cough | 6 |
| Appendicitis | 6 |
| Deep vein thrombosis | 6 |
| Oedema | 6 |
| Arrhythmia | 6 |
| Pyrexia | 6 |
| Gastrointestinal disorder | 5 |
| Upper respiratory tract infection | 5 |
| Neuralgia | 5 |
| Thrombosis | 5 |
| Chest discomfort | 5 |
| Angina pectoris | 4 |
| Arthralgia | 4 |
| Bell's palsy | 4 |
| Vomiting | 4 |
| Pyelonephritis | 4 |
| Ear infection | 4 |
| Blood iron decreased | 4 |
| Pancreatitis | 4 |
| Gastrooesophageal reflux disease | 4 |
| Eye inflammation | 4 |
| Neoplasm malignant | 4 |
| Bacterial infection | 4 |
| Bursitis | 4 |
| Tinnitus | 4 |
| Abdominal pain | 4 |
| Heart rate irregular | 3 |
| Paraesthesia | 3 |
| Herpes simplex | 3 |
| Aphthous ulcer | 3 |
| Arthritis | 3 |
| Diverticulitis | 3 |
| Skin infection | 3 |
| Diarrhoea | 3 |
| Heart rate increased | 3 |
| Gastritis | 3 |
| Trigeminal neuralgia | 3 |
| Hypotension | 3 |
| Abscess | 3 |
| Giant cell arteritis | 3 |
| Hernia | 3 |
| Circulatory collapse | 3 |
| Tachycardia | 3 |
| Pain | 3 |
| Muscle spasms | 3 |
| Anaemia | 3 |
| Condition aggravated | 3 |
| Cholecystectomy | 3 |
| Hypoaesthesia | 3 |
| Autoimmune disorder | 3 |
| Subdiaphragmatic abscess | 2 |
| Otitis media | 2 |
| Myocardial infarction | 2 |
| Fracture | 2 |
| Pharyngitis | 2 |
| Cardiac valve disease | 2 |
| Costochondritis | 2 |
| Cardiomyopathy | 2 |
| Deafness neurosensory | 2 |
| Haemorrhage urinary tract | 2 |
| Periarthritis | 2 |
| Blood sodium decreased | 2 |
| Respiratory tract infection | 2 |
| Anaphylactic reaction | 2 |
| Menstrual disorder | 2 |
| Breast cancer | 2 |
| Myalgia | 2 |
| Cholelithiasis | 2 |
| Nausea | 2 |
| Irritable bowel syndrome | 2 |
| Oligomenorrhoea | 2 |
| Ischaemic stroke | 2 |
| Dermal cyst | 2 |
| Constipation | 2 |
| Pericardial effusion | 2 |
| Aortic stent insertion | 2 |
| Cardiac arrest | 2 |
| Malaise | 2 |
| Stent placement | 2 |
| Meniere's disease | 2 |
| Meningitis | 2 |
| Amenorrhoea | 2 |
| Polycystic ovaries | 1 |
| Echocardiogram | 1 |
| Seasonal allergy | 1 |
| Internal haemorrhage | 1 |
| Parathyroid disorder | 1 |
| Interstitial lung disease | 1 |
| Coeliac disease | 1 |
| Intervertebral disc compression | 1 |
| Stenosis | 1 |
| Glaucoma | 1 |
| Tremor | 1 |
| Gout | 1 |
| Diabetes mellitus | 1 |
| Jejunal operation | 1 |
| Postmenopausal haemorrhage | 1 |
| Varicella zoster virus infection | 1 |
| Rectal cancer | 1 |
| Bronchiectasis | 1 |
| Shoulder operation | 1 |
| Conjunctival oedema | 1 |
| Superficial vein thrombosis | 1 |
| Cardiac operation | 1 |
| Thyroiditis | 1 |
| Lichen planus | 1 |
| Cardiac failure chronic | 1 |
| Lower limb fracture | 1 |
| Hypothyroidism | 1 |
| Aortic aneurysm | 1 |
| Pleurisy | 1 |
| Lung disorder | 1 |
| Polymyalgia rheumatica | 1 |
| Lung neoplasm malignant | 1 |
| Capillary leak syndrome | 1 |
| Balance disorder | 1 |
| Radiculitis brachial | 1 |
| Lymphoedema | 1 |
| Colitis | 1 |
| Cervix carcinoma | 1 |
| Seizure | 1 |
| Malignant melanoma | 1 |
| Adnexal torsion | 1 |
| Mantle cell lymphoma | 1 |
| Injury | 1 |
| Mast cell activation syndrome | 1 |
| Cardiac failure | 1 |
| Mastitis | 1 |
| Thyroid mass | 1 |
| Chemotherapy | 1 |
| Eczema | 1 |
| Hepatic steatosis | 1 |
| Type IV hypersensitivity reaction | 1 |
| Gastrointestinal carcinoma | 1 |
| Urosepsis | 1 |
| Mental disorder | 1 |
| Pelvic pain | 1 |
| Contusion | 1 |
| Cluster headache | 1 |
| Mitral valve incompetence | 1 |
| Infectious mononucleosis | 1 |
| Monoclonal B-cell lymphocytosis | 1 |
| Diabetic ketoacidosis | 1 |
| Motor neurone disease | 1 |
| Polymenorrhoea | 1 |
| Coronary artery bypass | 1 |
| Post-acute COVID-19 syndrome | 1 |
| Adrenal insufficiency | 1 |
| Prostate cancer | 1 |
| Cardiac pacemaker insertion | 1 |
| Pulmonary fibrosis | 1 |
| Hip arthroplasty | 1 |
| Dizziness | 1 |
| Benign prostatic hyperplasia | 1 |
| Arthritis viral | 1 |
| Nasopharyngitis | 1 |
| Renal pain | 1 |
| Hyperglycaemia | 1 |
| Rheumatoid arthritis | 1 |
| Atrial flutter | 1 |
| Secretion discharge | 1 |
| Autoimmune arthritis | 1 |
| Shortened cervix | 1 |
| Craniocerebral injury | 1 |
| Abortion spontaneous | 1 |
| Nervous system disorder | 1 |
| Sleep disorder | 1 |
| Bradycardia | 1 |
| Colitis ulcerative | 1 |
| Blood glucose increased | 1 |
| Suicidal ideation | 1 |
| Food poisoning | 1 |
| Swelling | 1 |
| Dehydration | 1 |
| Testicular pain | 1 |
| Appendicitis perforated | 1 |
| Thyroid disorder | 1 |
| Cardiac failure congestive | 1 |
| Thyroidectomy | 1 |
| Ovarian cyst ruptured | 1 |
| Tinea infection | 1 |
| Ovarian hyperstimulation syndrome | 1 |
| Atelectasis | 1 |
| Delirium | 1 |
| Emphysema | 1 |
| Depression | 1 |
| Umbilical hernia | 1 |
| Breast swelling | 1 |
| Amnesia | 1 |
| Panic attack | 1 |
| Insomnia | 1 |
| Dermatitis bullous | 1 |
| Fibromyalgia | 1 |
| Nephritis | 1 |
| Joint injury | 1 |
| Vitreous floaters | 1 |
| Lacunar stroke | 1 |
| Wisdom teeth removal | 1 |
| Lethargy | 1 |
| Back pain | 1 |
| Leukaemia | 1 |
| **Total** | **1,109** |
| *** Terms not mapped to MedDRA**  Free text responses that indicated the participant was ‘undergoing tests’ or ‘waiting to be diagnosed’ were termed ‘awaiting diagnosis’. Responses stating a ‘vaccine reaction’ or ‘problem caused by the vaccine’ without sufficient detail to map to a diagnosis were termed ‘vaccine reaction self-declared’. Free text diagnoses that were unable to be specifically classified in accordance with MedDRA were classified as ‘other’; majority of these were ‘unknown/unexplainable/no diagnosis’. | |
