# Supplementary figures and images for: Safety of Four COVID-19 Vaccines across Primary Doses 1, 2, 3 and Booster: A Prospective Cohort Study of Australian Community Pharmacy Vaccinations
Source: Vaccines (Basel). 2022 Nov 25;10(12):2017. doi: 10.3390/vaccines10122017 (PMC9786585; doi:10.3390/vaccines10122017)

Figure S1. Flow-chart of the study.

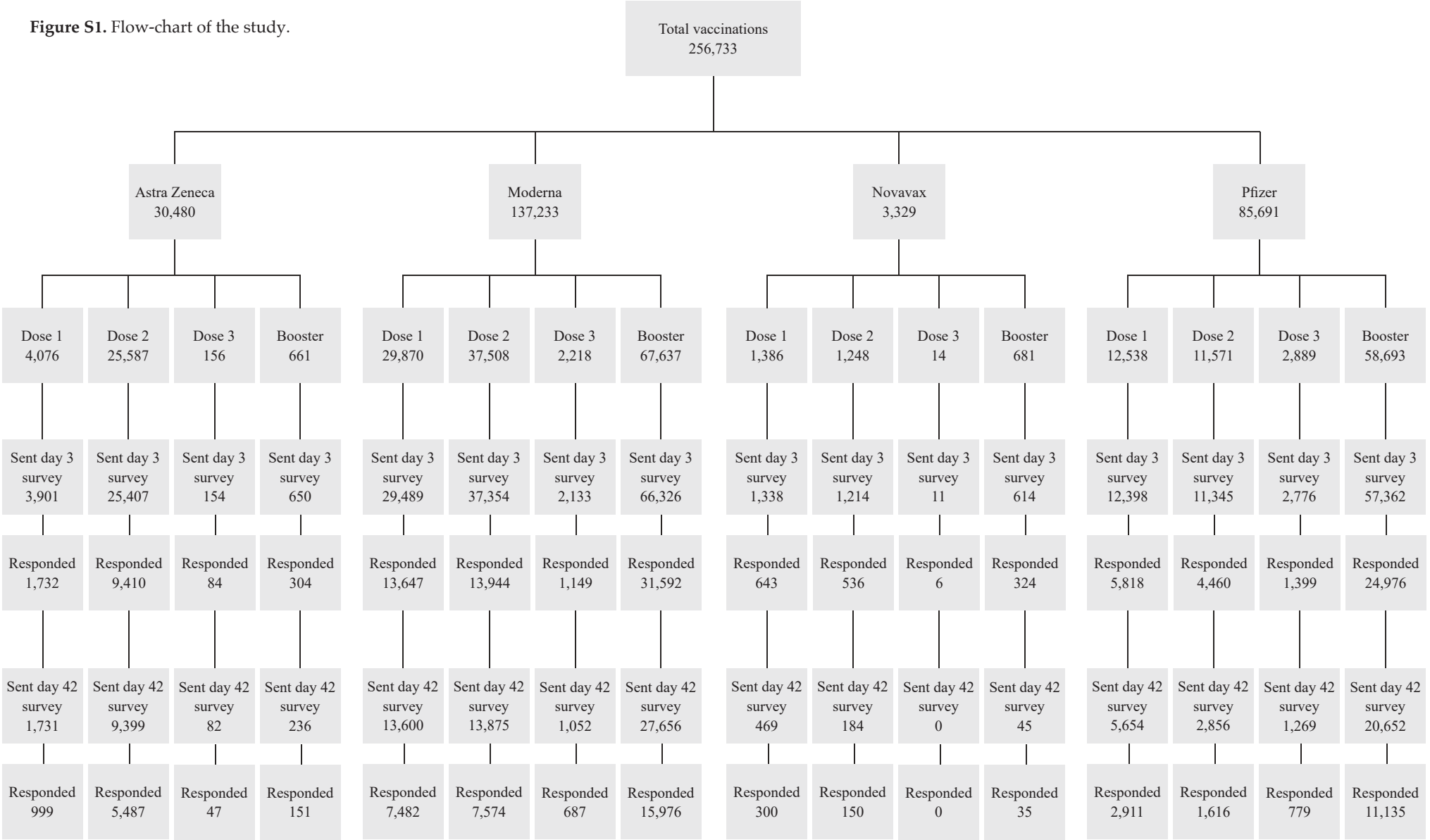

Supplement: Supplementary file 1 [file vaccines-10-02017-s001.zip › Figure S1- Flowchart.pdf]
